# Persistent Human T‐Lymphotropic Virus Type 1 (HTLV‐1) Infection in the Placenta of Pregnant Women

**DOI:** 10.1002/jmv.70585

**Published:** 2025-09-05

**Authors:** Gabriela Prates, Xiaoyi Li, Victor Folgosi, George Souza, Sandy Teixeira, Carlos Apoliano, Fernanda Grassi, Jacielma Freire, Jerusa Smid, Michel E. Haziot, Rosa Maria N. Marcusso, Augusto C. P. de Oliveira, Hongjie Chen, Tatiane Assone, Yuyang Tang, Guochun Jiang, Jorge Casseb

**Affiliations:** ^1^ Laboratory of Dermatology and Immunodeficiencies, Department of Dermatology University of São Paulo Medical School São Paulo Brazil; ^2^ UNC HIV Cure Center University of North Carolina Chapel Hill North Carolina USA; ^3^ Institute of Global Health & Infectious Diseases University of North Carolina Chapel Hill North Carolina USA; ^4^ Gonçalo Moniz Institute Oswaldo Cruz Foundation (FIOCRUZ) Salvador Bahia Brazil; ^5^ Climério de Oliveira Maternity Hospital Bahia Federal University of Bahia (UFABA) Bahia Brazil; ^6^ Institute of Infectious Diseases “Emilio Ribas” (IIER) de São Paulo São Paulo SP Brazil; ^7^ Departamento de Medicina Legal, Bioética, Medicina do Trabalho e Medicina Física e Reabilitação Faculdade de Medicina da Universidade de São Paulo São Paulo Brazil; ^8^ Department of Biochemistry and Biophysics University of North Carolina Chapel Hill North Carolina USA

**Keywords:** HERV‐W1 Env, HTLV‐1, IFNβ, placenta, spontaneous abortions, syncytin‐1

## Abstract

Mother‐to‐child transmission (MTCT) is the primary route of human T‐lymphotropic virus type 1 (HTLV‐1) infection. Although formula feeding reduces breastfeeding‐associated transmission, MTCT still occurs, implicating pregnancy or delivery as key transmission windows. In this study, placental tissues from nine HTLV‐1–positive mothers were analyzed using DNA/RNAscope, revealing low HTLV‐1 DNA and RNA levels and a low RNA/DNA ratio, consistent with latent infection in the placenta and potentially explaining the low MTCT rate. Elevated interferon (IFN)‐β levels were observed in infected placentas compared to seronegative controls, while IFNα, IFNγ, and IFITM expression remained unchanged. Concurrently, sustained IFNβ expression in infected placentas suggests its dual roles in HTLV‐1 pathogenesis: suppressing viral replication while potentially disrupting placental homeostasis through chronic inflammation. In vitro modeling using BeWo cells or primary trophoblasts cocultured with HTLV‐1‐infected MT‐2 cells demonstrated syncytin‐1‐mediated viral entry, confirmed by HTLV‐1 p19 detection in both trophoblasts. Of note, HTLV‐1 transmission was abolished by a syncytin‐1‐specific fusion inhibitor HRB1, underscoring syncytin‐1's essential role in cell‐to‐cell transmission of HTLV‐1. Thus, this study identifies syncytin‐1 as a therapeutic target to block vertical transmission and highlights the need to balance antiviral responses with placental integrity in HTLV‐1 management.

## 1 Introduction

Mother‐to‐child transmission (MTCT) of human T‐lymphotropic virus type 1 (HTLV‐1) occurs predominantly via breastfeeding, accounting for 12%–25% of HTLV‐1 infection [1, 2, 3]. However, even among formula‐fed infants, a residual transmission rate of 2.4%–3.6% persists [4], suggesting intrauterine transmission or delivery‐associated exposure as critical routes. Emerging evidence implicates HTLV‐1 in placental dysfunction: a regional cohort of > 130 000 pregnant women identified 153 HTLV‐1‐positive mothers, exhibited high miscarriage rates of 22%–30%, with 6.7% of spontaneous abortions occurring in the first trimester [5]. Notably, placental inflammation underlies 50% of preterm deliveries [6], yet the mechanisms by which HTLV‐1 disrupts placental homeostasis remain poorly understood.

HTLV‐1 establishes persistent, low‐level infection in placental trophoblasts, characterized by minimal viral DNA and RNA, indicative of restricted replication. Viral persistence may perturb the placental environment through interferon (IFN) pathway activation. While IFN cascades typically mediate antiviral defense, chronic inflammation can disrupt embryo implantation, angiogenic signaling, and fetal development. In HTLV‐1‐associated myelopathy (HAM), dysregulated pro‐inflammatory cytokine networks exacerbate tissue damage [7, 8, 9, 10, 11], raising the possibility that similar mechanisms compromise the tolerogenic state essential for pregnancy [12, 13].

The syncytiotrophoblast layer serves as a major barrier to intrauterine transmission [14, 15]. Syncytin, a retroviral envelope protein highly expressed in trophoblast cells, facilitates syncytiotrophoblast formation through fusogenic activity. Decreased syncytin expression has been observed in the placenta affected by fetal growth restriction [16, 17, 18, 19]. HTLV‐1 viral protein expression, including Tax and basic leucine zipper factor (HBZ), induces inflammation in multiple systems in HTLV‐1‐infected women [20]. Notably, Tax and HBZ also play critical roles in HTLV‐1 infection and the survival and transformation of leukemic cells in the peripheral blood [21, 22].

In response to the initial stage of viral infection, interferon‐induced transmembrane proteins (IFITMs) prevent viral fusion with cellular membranes [23, 24]. Elevated IFITM levels impair syncytiotrophoblast layer formation and inhibit syncytin‐mediated fusion. This may contribute to pregnancy complications observed in congenital infections and other IFN‐associated inflammatory conditions [24]. Th1 cytokines induce immune rejection, thereby disrupting pregnancy, while Th2 cytokines suppress Th1 activity and promote fetal‐maternal immune tolerance, essential for maintaining gestation [25]. For example, IFNγ inhibits extravillous trophoblast (EVT) invasion—a process critical for placental development—by inducing apoptosis in these cells [26].

We hypothesize that HTLV‐1 infiltration into the placenta triggers inflammatory cascades that impair gestational progression. This imbalance may not only facilitate vertical transmission but also heighten risks of miscarriage, preterm birth, and fetal compromise in mothers living with HTLV‐1 (MLHTLV‐1). Our study investigates the interplay between HTLV‐1 persistence, syncytin‐1‐dependent entry, and IFN‐driven inflammation in placental dysfunction, offering insights into mechanisms linking viral infection to adverse pregnancy outcomes.

## 2 Materials and Methods

### 2.1 Human Research Participants

Before April 2022, Brazil lacked widespread prenatal HTLV‐1 screening. Participants were recruited from the Institute of Infectology Emílio Ribas, which hosts the largest HTLV‐1 outpatient cohort globally, and Climerio de Oliveira Maternity Hospital, a referral center for high‐risk pregnancies in Salvador, Bahia. The latter performs approximately 1300 deliveries annually and identifies HTLV‐1 in an average of 10.8 pregnancies per year among seropositive mothers. Nine HTLV‐1‐seropositive mothers were enrolled between December 2019 and March 2024, with recruitment paused from February 2020 to February 2022 due to the COVID‐19 pandemic. A control group of nine HTLV‐1‐seronegative mothers was included, all aged ≥ 18 years (range: 19–42 years), with full‐term pregnancies, negative serology for hepatitis B/C, rubella, and toxoplasmosis, and no history of smoking or prolonged membrane rupture (> 8 h).

No medical guidelines currently specify a preferred delivery mode for HTLV‐1‐seropositive mothers. Delivery mode (vaginal or cesarean) was determined through shared decision‐making between patients and clinicians. As no antivirals exist to suppress HTLV‐1 replication, none of the seropositive mothers received antiretroviral therapy. The complete clinical histories of all 18 participants, including one seropositive individual treated with methylprednisolone for HTLV‐1‐associated myelopathy (HAM), are detailed in Supporting Information S1: Table 1. The study protocol was approved by the Ethics Committees of the Institute of Tropical Medicine/Faculty of Medicine (University of São Paulo; CAAE: 43288720.4.1001.0068), the Institute of Infectology Emilio Ribas (CAAE: 43288720.4.3003.5543), and Climerio de Oliveira Maternity Hospital (CAAE: 43288720.4.3001.0061). Written informed consent was obtained from all participants.

### 2.2 Peripheral Blood Mononuclear Cells (PBMCS) HTLV‐1 DNA Load Quantification

Quantification of HTLV‐1 DNA load in PBMCs isolated from the participants was performed by real‐time PCR using primers and probes targeting the *pol* gene. For HTLV‐1 DNA quantitation, the forward primer SK110 (5′‐CCCTACAATCCAACCAGCTCAG‐3′; nucleotides 4758–4779, GenBank accession no. J02029) and reverse primer SK111 (5′‐GTGGTGAAGCTGCCATCGGGTTTT‐3′; nucleotides 4943–4920) were used. The internal HTLV‐1 TaqMan probe (5′‐CTTTACTGACAAACCCGACCTACCCATGGA‐3′) was designed using Oligo software (version 4; National Biosciences, Plymouth, MI, USA) and Primer Express (Perkin‐Elmer Applied Biosystems, Boston, MA, USA) and verified by GenBank search. The probe, located between positions 4829 and 4858 of the HTLV‐1 genome, carried a 5′ reporter dye FAM (6‐carboxyfluorescein) and a 3′ quencher dye TAMRA (6‐carboxytetramethylrhodamine). For quantification of the human albumin gene, the forward primer Alb‐S (5′‐GCTGTCATCTCTTGTGGGCTGT‐3′), reverse primer Alb‐AS (5′‐AAACTCATGGGAGCTGCTGGTT‐3′), and albumin TaqMan probe (5′‐FAM‐CCTGTCATGCCCACACAAATCTCTCC‐TAMRA‐3′) were used.

Each 50 μL PCR reaction for HTLV‐1 or albumin amplification contained 10 μL DNA extract; primers SK110/SK111 or Alb‐S/Alb‐AS (200 nM each); 100 nM HTLV‐1 or albumin probe; 200 nM each of dATP, dCTP, and dGTP; 400 nM dUTP; 5 mM MgCl_2_; 0.5 U uracil DNA glycosylase; 1.25 U Platinum Taq DNA polymerase; and 1× PCR buffer. Thermal cycling conditions were: 50°C for 2 min; 95°C for 10 min; followed by 45 cycles of 95°C for 15 s and 65°C for 1 min. Amplification and data acquisition were performed on an iCycler Sequence Detector System (Bio‐Rad, Hercules, CA, USA).

MT‐2 cells, containing one copy of HTLV‐1 DNA per cell (kindly provided by Dana Gallo, Viral and Rickettsial Disease Laboratory, Richmond, CA, USA), were used as the positive control and serially diluted to final concentrations of 10^5^, 10^4^, 10^3^, and 10^2^ cells. For the albumin control, serial dilutions of HTLV‐1‐negative DNA were prepared in parallel. Standard curves for both HTLV‐1 and albumin were accepted only when the slope ranged from −3.74 to −3.32 (corresponding to PCR efficiencies of 85%–100%) and the coefficient of correlation (*r* ^2^) exceeded 0.98. Assays were repeated if the difference between duplicate measurements of HTLV‐1 or albumin DNA copy numbers was greater than 30%. Normalized HTLV‐1 proviral load was calculated as:

$$\mathrm{Proviral}\;\mathrm{load}\;(\mathrm{copies}/10^{6}\;\mathrm{PBMCs})=\frac{\mathrm{HTLV}-1\;\mathrm{DNA}\;\mathrm{average}\;\mathrm{copy}\;\mathrm{numbe}r}{\mathrm{Albumin}\;\mathrm{DNA}\;\mathrm{average}\;\mathrm{copy}\;\mathrm{number}}\times 2\times 10^{6}$$

### 2.3 Placenta Collection and Preparation

Cotyledons (approximately 1 cm^2^ placental tissue) were excised from a randomly chosen central area immediately after placental withdrawal. They were then transported in either formaldehyde solution or DMEM/F12 (Gibco, CA, USA). To eliminate residual blood, samples were rinsed with a saline solution. A 50 mg section was obtained from both the deciduous (maternal side) and the villus (fetal side) after being fixed in formalin and embedded in paraffin.

### 2.4 HTLV‐1 DNA or RNA Scope and CK7 Immunostaining

Paraffin‐embedded placental blocks were sectioned into 6 μm slices, deparaffinized in xylene, and rehydrated through graded ethanol. For RNAscope assays, slides were processed using the RNAscope 2.5 HD Detection Reagent‐Red (ACD) following the manufacturer's protocol. For DNA detection, slides were pretreated with RNase A (100 μg/mL) to remove RNA, followed by Protease IV digestion (ACD) to expose target DNA. Slides were then incubated in RNAscope Hydrogen Peroxide (10 min, room temperature [RT]) and boiled (100°C) in 1×RNAscope Target Retrieval Buffer (30 min). After ethanol dehydration, slides underwent Protease Plus treatment (40°C, 30 min). A hydrophobic barrier was applied to confine reagents, and DNA was denatured (60°C, 10 min). Probes for HTLV‐1 RNA (HBZ: ACD #472861; Tax: ACD #472871) or DNA (gag‐pol: ACD #495061) were hybridized overnight at 40°C. Signals were visualized using RED‐A/RED‐B chromogens. For dual immunohistochemistry, slides were blocked with 15% goat serum and 1% Fc receptor blocking reagent (BioLegend) in TBST (45 min, RT). Anti‐CK7 antibody (Abcam, 1:200 in TBST) was applied overnight at 4°C. After TBST washes, biotinylated goat anti‐mouse IgG (1:800, 30 min, RT) and streptavidin‐HRP (1:500, 30 min, RT) were sequentially incubated. Staining was developed with ImmPACT DAB Peroxidase Substrate (Vector Laboratories, SK‐4105; 2 min, RT) and stopped with distilled water.

### 2.5 RNA Extraction From Paraffined Tissues

Formalin‐fixed, paraffin‐embedded (FFPE) tissues were sectioned into 5–20 μm‐thick slices using a microtome and collected into 1.7 mL microcentrifuge tubes. RNA was isolated from sections using the PureLink FFPE Total RNA Isolation Kit (Thermo Fisher Scientific, Foster City, CA) following the manufacturer's protocol. RNA concentration and purity were assessed by measuring the A260/A280 absorbance ratio using a spectrophotometer (DeNovix). For downstream cDNA synthesis, RNA was standardized to a concentration of 50 ng/μL.

### 2.6 cDNA Synthesis

RNA samples adjusted at 50 ng/μL were treated with DNase I, then incubated with Random primers at 300 ng/μL for 10 min. cDNA synthesis was performed in a 40 μL reaction mixture containing RT IV buffer (Invitrogen), deoxynucleoside triphosphates (dNTPs), RNase inhibitor (Invitrogen), reverse transcriptase, and water. cDNA was used for droplet digital polymerase chain reaction assay with triplicate wells.

### 2.7 Droplet Digital Polymerase Chain Reaction

Droplet digital polymerase chain reactions (ddPCR) consisted of a 20 μL mixture per well containing 2× ddPCR Probe Supermix (Bio‐Rad, Hercules, CA, USA), 1.1 μL of 20× target primers 900 nM/250 nM of syncytin‐1, IFNα, IFNβ, IFNγ, or IFITM probe (Applied Biosystems), 1.1 μL of water, and 8.8 μL of cDNA. Droplets were generated in the QX‐200 droplet generator (Bio‐Rad) according to the manufacturer's instructions. Droplets were transferred to a 96‐well PCR plate (Eppendorf, Hamburg, Germany) and the subsequent PCR amplification was performed in the thermal cycler C1000 Touch Thermal Cycler(Bio‐Rad) with the cycling parameters; 95°C for 10 min Ramp 2°C/s, 94°C for 30 s Ramp 2°C/s, 57°C for 1 min Ramp 2°C/s, Goto 2, 45X, 98°C for 10 min, and then incubated overnight at 4°C. Droplets were read in the QX200 droplet reader (Bio‐Rad) and visualized with QuantaSoft version 1.6.6 (Bio‐Rad). Each sample was analyzed in triplicate, and a no‐template control (NTC) was added for each reaction round. Only wells containing ≥ 1000 droplets were accepted for further analysis. Based on the background in the NTC, a threshold was set to define positive droplets and calculate the absolute concentration for each sample.

### 2.8 Flow Cytometry

MT‐2 cells were fixed and permeabilized using BD Cytofix/Cytoperm Fixation/Permeabilization Kit (BD bioscience). Then, cells were incubated with anti‐ASCT1 antibody (1:100, ATLAS Antibodies) or anti‐ASCT2 antibody (1:100, Cell Signaling) for 30 min at 4°C. Lastly, cells were incubated with anti‐rabbit FITC antibody (Abcam) for 30 min at 4°C. ASCT1 or ASCT2 expression was quantified by FITC expression using flow cytometry, and the data were analyzed using FlowJo Software.

### 2.9 Cell Culture and Coculture of MT‐2 Cells With BeWo Cells or Primary Cytotrophoblasts (PCTs)

BeWo cells (human choriocarcinoma‐derived) were maintained in ATCC‐formulated F‐12K medium. PCTs were obtained from ScienCell Research Laboratories (Cat# 7120) and maintained in trophoblast medium (ScienCell Research Laboratories). MT‐2 cells were cultured in RPMI‐1640 medium. All media contained 10% heat‐inactivated fetal bovine serum (FBS), 2 mM l‐glutamine, 10 mM HEPES, and 1% antibiotics (10 000 U/mL penicillin, 10 mg/mL streptomycin). All cell lines were incubated at 37°C under 5% CO_2_ and 95% humidity. Media were refreshed every 2–3 days. BeWo cells or PCTs (1.6 × 10^5^ per well) were seeded into 6‐well plates and allowed to adhere for 24 h. Cells were then treated for 24 h with forskolin (20 μM or 50 μM for BeWo; 50 μM or 100 μM for PCTs; Sigma‐Aldrich) or left untreated. Following treatment, cells were washed with PBS (Gibco) to remove forskolin, and fresh medium was added. MT‐2 cells were incubated with mitomycin C (20 μg/mL; Sigma‐Aldrich) for 1 h at 37°C. For the HRB1 treatment group, target cells and MT‐2 cells were pretreated separately with HRB1 (10 μg/mL) for 1 h before coculture. HRB1‐pretreated or untreated MT‐2 cells were then added to BeWo cells or PCTs at a 1:10 ratio (MT‐2:target) and cocultured for 48 h. After coculture, non‐adherent MT‐2 cells were removed by gentle PBS washing, and fresh medium was added. BeWo cells or PCTs were cultured for an additional 72 h. Finally, BeWo cells or PCTs were detached using 0.25% trypsin‐EDTA (Gibco; 5 min, 37°C). Trypsin activity was neutralized with complete medium, and cells were pelleted by centrifugation (300*g*, 5 min), followed by one PBS wash [7].

### 2.10 HTLV‐1 p19 and Syncytin‐1 Detection by Western Blot

Total cell proteins from BeWo cells or PCTs were extracted with RIPA lysis buffer (Thermo Fisher Scientific), containing a 1× protease and phosphatase inhibitor cocktail (Cell Signaling). Protein expression was assessed using the following antibodies: anti‐HTLV type I p19 clone 45/6.11.1.3 (1:2000, ZeptoMetrix), syncytin‐1 polyclonal antibody (1:3000, Bioss), and β‐actin antibody (1:2500, Cell Signaling).

### 2.11 Statistical Analysis

Statistical analyses were performed using Prism software (GraphPad, version 9). Parametric data were analyzed with *t*‐test, and non‐parametric data with the Mann–Whitney test. *p* values less than 0.05 were considered statistically significant.

## 3 Results

### 3.1 Characteristics of Study Participants

Nine HTLV‐1‐seronegative mothers and nine HTLV‐1‐seropositive mothers were included in the study. The mean maternal age at delivery differed significantly between the two groups: 24.4 years in the seronegative group versus 32.1 years in the seropositive group (*p* = 0.04). No significant difference was observed in infant sex distribution: 44.4% (4/9) of infants born to seronegative mothers were male, compared to 66.6% (6/9) in the seropositive group (*p* = 0.63). Delivery modes varied markedly, with 88.8% of seronegative mothers having vaginal deliveries versus 33.3% in the seropositive group (*p* = 0.02). Reproductive history differed significantly: 88.8% of seronegative mothers reported no prior pregnancies, whereas 88.8% of seropositive mothers had previous pregnancies, including those resulting in spontaneous abortions (*p* = 0.003). Syphilis co‐infections and gestational diabetes were exclusively observed in the seropositive group. Most seropositive mothers were asymptomatic (88.8%), with one case of HTLV‐1‐associated myelopathy (HAM). Notably, 44.4% of seropositive mothers reported prior spontaneous abortions, compared to 11.1% in the seronegative group; however, this difference was not statistically significant (*p *= 0.29) (Table 1).

**Table 1:** Clinical characteristics of HTLV‐1‐uninfected mothers and MLHTLV‐1.

	HTLV‐1 [−]	HTLV‐1 [+]	OR [95% CI]	*p* value
Total *n*. 18	9 (50.0%)	9 (50.0%)		
Mother's age at delivery (years)				
Mean (±SD)	24.4 (6.4)	32.1 (8.2)		0.04
Baby sex				
Male	4 (44.4%)	6 (66.6%)	0.4 [0.07–2.3]	0.63
Female	5 (55.5%)	3 (33.3%)		
Delivery				
Vaginal	8 (88.8%)	3 (33.3%)		0.02
Cesarean without labor	0	5 (55.5%)		
Cesarean with labor	1 (11.1%)	1 (11.1%)		
Previous pregnancy				
No	8 (88.8%)	1 (11.1%)	64.0 [3.7–761.6]	0.003
Yes	1 (11.1%)	8 (88.8%)		
Coinfections/comorbidities				
No	9 (100.0)	6 (66.6%		0.06
Syphilis	0	1 (11.1%)		
Gestational diabetes	0	2 (22.2%)		
HTLV‐1‐clinical outcome	—	(5.4%)		
Asymptomatic	—	8 (88.8%)		—
HAM	—	1 (11.1%)		
Previous spontaneous abortions		4 (44.4%)		
No	8 (88.8%)	5 (55.5%)	6.4 [0.57–86.3]	0.29
Yes	1 (11.1%)	4 (44.4%)		
Median HTLV‐1 proviral load (copies/10^6^ PBMCs)	0	36.9 (50%)		

*Note:* A *t*‐test was used for comparing means, the Fisher test for bivariate variables, and the *χ* ^2^ trend test for trivariate. We did not proceed with multivariate analysis due to the presence of variables with a *p* value > 0.02.

### 3.2 Detection of HTLV‐1 DNA/RNA and Evidence of Latent Infection in Placental Tissues

**Figure 1:**
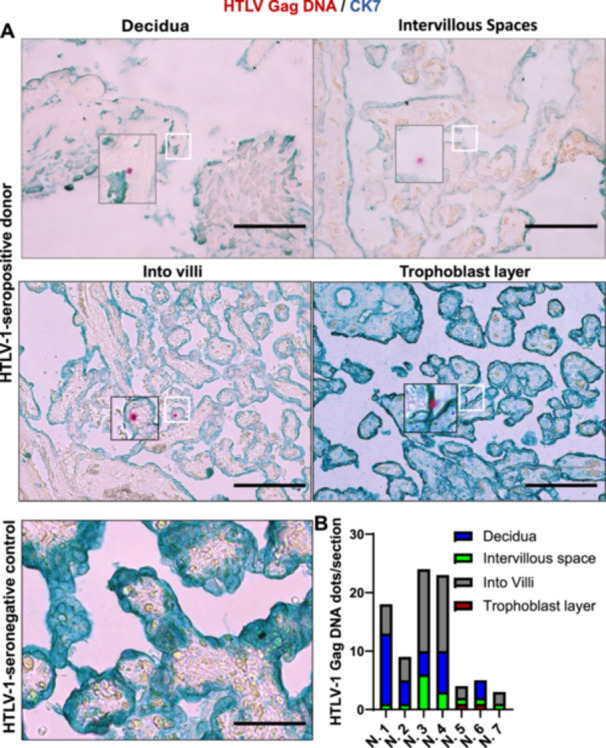
The detection of HTLV‐1 gag DNA in the placenta. (A) HTLV‐1 gag DNA in different placental regions was visualized using in situ RNAscope hybridization in combination with immunofluorescence assay of trophoblast marker protein CK7 (Scale bar, 250 μm). Red, HTLV‐1 RNA; Green, trophoblast marker CK7. (B) HTLV‐1 gag DNA was quantitated in the decidua, intervillar space, villi, and trophoblasts (*n* = 7).

**Figure 2:**
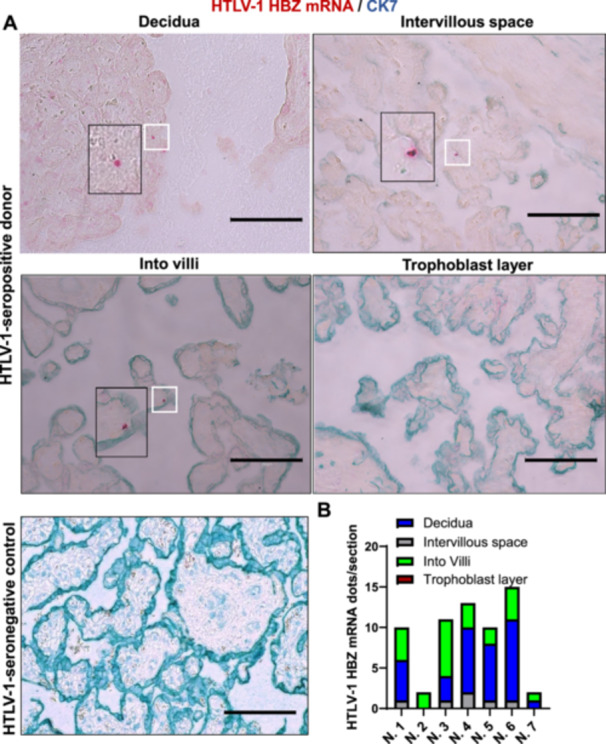
The detection of HTLV‐1 HBZ RNA in the placenta. (A) HTLV‐1 HBZ RNA in different placental regions was visualized using in situ DNAscope hybridization in combination with immunofluorescence assay of trophoblast marker protein CK7 (Scale bar, 250 μm). Red, HTLV‐1 RNA; Green, trophoblast marker CK7. (B) HTLV‐1 HBZ RNA was quantitated in the decidua, intervillar space, villi, and trophoblasts (*n* = 7).

**Figure 3:**
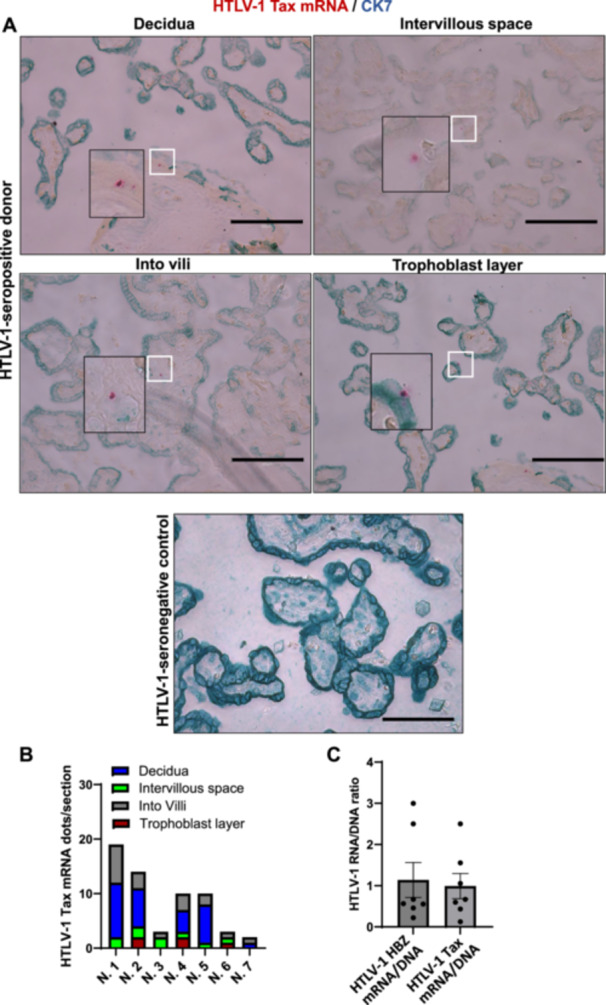
The detection of HTLV‐1 Tax RNA in the placenta. (A) HTLV‐1 Tax RNA in different placental regions was visualized using in situ DNAscope hybridization in combination with immunofluorescence assay of trophoblast marker protein CK7 (Scale bar, 250 μm). Red, HTLV‐1 RNA; Green, trophoblast marker CK7. (B) HTLV‐1 HBZ RNA was quantified in the decidua, intervillar space, villi, and trophoblasts (*n* = 7). (C) The ratio of HTLV‐1 RNA/DNA in the placenta.

Term placental tissues from HTLV‐1‐seropositive mothers (*n* = 7) were analyzed using highly sensitive molecular methods. As reported previously [27], DNA in situ hybridization (DNAISH) and RNA in situ hybridization (RNAISH) techniques were adapted and refined here as HTLV‐1‐specific DNAscope and RNAscope assays to detect viral nucleic acids in different placental tissues. Co‐staining with cytokeratin 7 (CK7), a trophoblast biomarker [28], enabled precise localization of viral nucleic acids within placental sub‐regions. DNAscope assays detected HTLV‐1 gag DNA in all seropositive samples (7/7), with low abundance (maximum 24 dots per section) and regional variability (Figure 1A). Viral DNA localized predominantly to placental villi and decidua but was also observed in intervillous spaces and trophoblast layers (Figure 1B). No signal was detected in HTLV‐1‐seronegative controls (Figure 1A). RNAscope assays detected HTLV‐1 HBZ mRNA in all HTLV‐1‐seropositive mothers, albeit at low levels. HTLV‐1 HBZ mRNA localized specifically to the decidua, intervillous spaces, and intravilli but was absent in trophoblasts (Figure 2A,B). In contrast, HTLV‐1 Tax mRNA was observed in decidua, intervillous spaces, intravilli, and sporadically in trophoblasts, though trophoblast detection occurred only in three seropositive mothers (Figure 3A,B). Both transcripts exhibited low abundance (maximum 24 dots per section) and regional variability, mirroring the distribution pattern of HTLV‐1 *gag* DNA, which was predominantly localized to placental villi and decidua. These findings demonstrate that HTLV‐1 DNA/RNA localizes to placental villi, either within the trophoblast layers surrounding the villi or subjacent to these layers. Quantification of the viral RNA/DNA ratio revealed fewer than one viral RNA transcript per viral DNA across placental sub‐regions (Figure 3C). Low transcriptional activity, evidenced by the RNA/DNA ratio < 1, strongly suggests that HTLV‐1 persists in a latent state within the placenta.

### 3.3 IFNβ Upregulation in the HTLV‐1‐Infected Placenta

**Figure 4:**
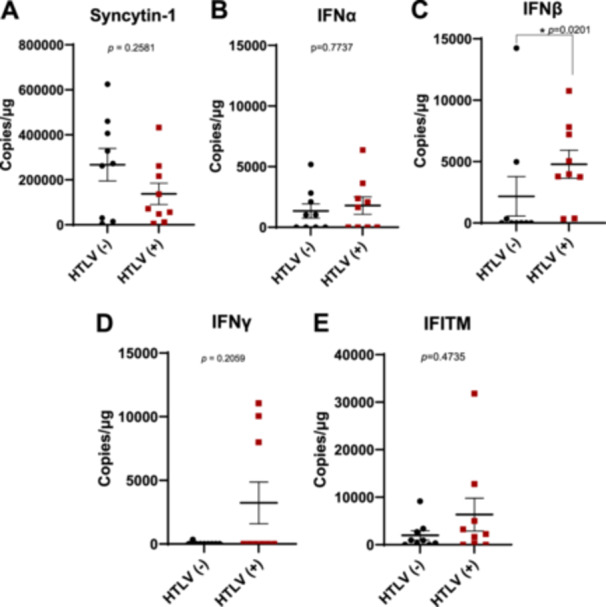
IFN*β* enrichment in the placenta from HTLV‐1‐infected mothers. The expression of (A) syncytin‐1, (B) IFNα, (C) IFNβ, (D) IFNγ, and (E) IFITM in placental tissues from HTLV‐1‐seropositive and HTLV‐1‐seronegative mothers was analyzed by ddPCR (*n* = 9).

Elevated type I IFN signaling during pregnancy is associated with adverse outcomes, including intrauterine growth restriction, preterm birth, and fetal demise [29]. IFN‐induced interferon‐induced transmembrane proteins (IFITMs), a family of viral entry restriction factors, are implicated in these complications by impairing syncytiotrophoblast formation and inhibiting syncytin‐1‐mediated cell fusion [23]. Excessive IFITM activity may thus contribute to placental dysfunction during congenital infections or IFN‐driven pathologies. HTLV‐1 placental infection may trigger local immune responses, potentially disrupting syncytiotrophoblast layer formation through inflammation, with implications for maternal and fetal health. Syncytins, fusogenic proteins critical for placental development, are central to this process. To investigate these, we quantified mRNA levels of human endogenous retrovirus‐W1 (HERV‐W1) syncytin‐1, IFNα, IFNβ, IFNγ, and IFITM in placental samples from nine HTLV‐1‐seropositive and nine HTLV‐1‐seronegative mothers. Placental tissues from seropositive mothers showed significantly higher IFNβ expression compared to seronegative controls (*p *= 0.0201) (Figure 4C). However, no significant differences were observed in HERV‐W1 syncytin‐1, IFNα, IFNγ, or IFITM expression between seropositive and seronegative groups (Figure 4A,B,D,E). Taken together, these results suggest that HTLV‐1 infection selectively upregulates IFNβ, potentially driving placental inflammation without triggering a broader IFN‐I signaling pathway.

### 3.4 Previous Spontaneous Abortion (SAB) Cases Among MLHTLV‐1 Were not Associated With the Expression of HERV‐W1 Syncytin‐1, IFNα, IFNβ, IFNγ, and IFITM

**Figure 5:**
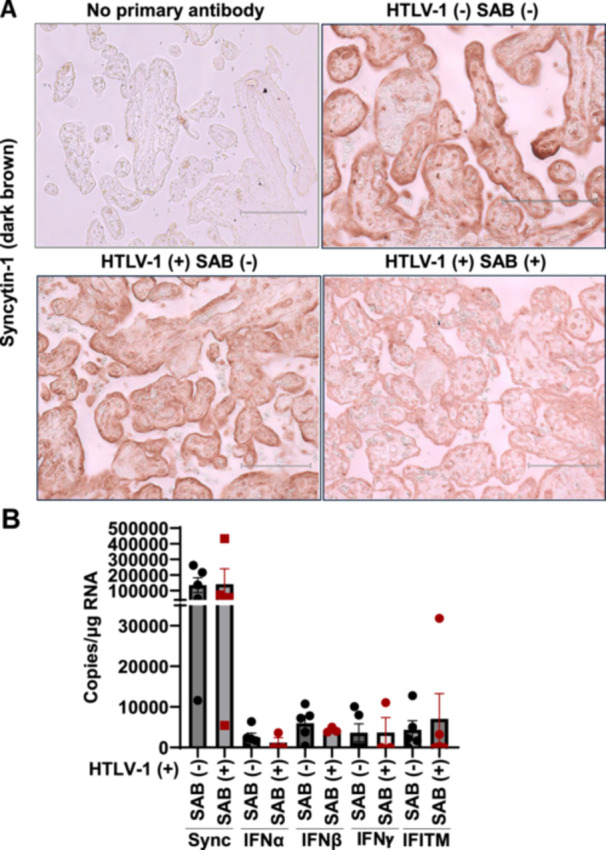
Previous spontaneous abortion (SAB) in HTLV‐1–infected mothers was not associated with placental expression of syncytin‐1, IFN‐β, IFN‐γ, or IFITM. (A) Syncytin‐1 in the human placenta was visualized by immunohistochemistry in the trophoblast border (Term placenta villi at 36–41 weeks of pregnancy) in the dark brown. Scale bar, 200 μm. (B) The expression of syncytin‐1, IFNα, IFNβ, IFNγ, or IFITM in the placenta from HTLV‐1‐infected women with or without previous SAB was measured by ddPCR.

A high prevalence of prior SAB was observed among HTLV‐1‐seropositive mothers (44.4%, 4/9) compared with seronegative controls (Table 1), suggesting a possible association between HTLV‐1 infection and adverse pregnancy outcomes. Placental HERV‐W1 syncytin‐1 expression, assessed by immunohistochemistry, showed no marked difference between HTLV‐1–seropositive mothers with a history of SAB and those without (Figure 5A). Similarly, syncytin‐1 expression measured by ddPCR did not differ significantly between these two groups (Figure 5B). Excessive IFITM activity has been proposed to impair syncytiotrophoblast formation and syncytin‐1–mediated fusion, potentially contributing to pregnancy complications in congenital infections or IFN‐driven pathologies [29]. To investigate this, we compared IFNα, IFNβ, IFNγ, and IFITM levels between HTLV‐1–seropositive mothers with and without prior SAB. No significant differences in IFITM, IFNα, or IFNβ expression were observed between HTLV‐1–seropositive mothers with a history of SAB and those without (Figure 5B).

### 3.5 Syncytin‐1 may Mediate HTLV‐1 Infection of Placental Trophoblasts

**Figure 6:**
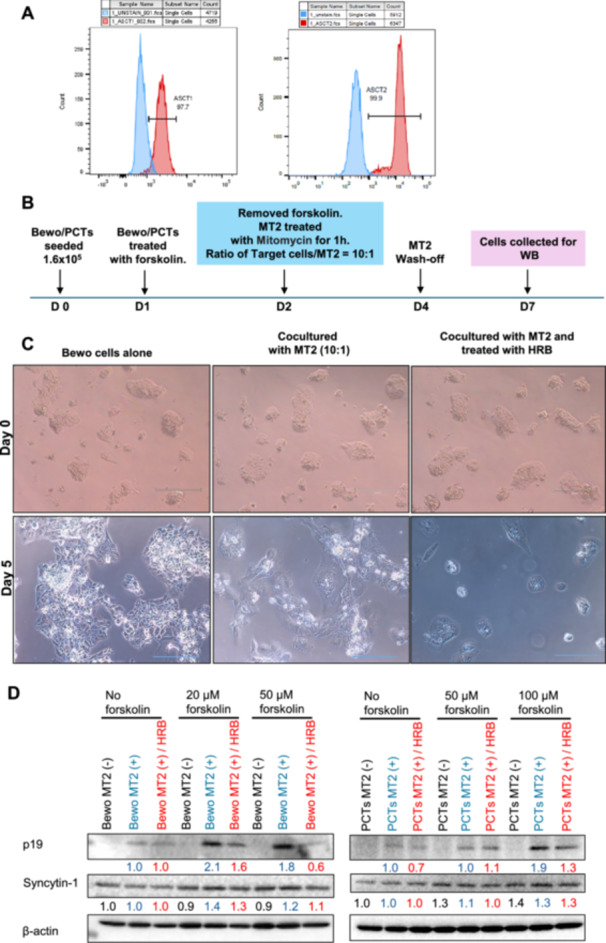
Syncytin‐1 may mediate HTLV‐1 infection of placental trophoblasts. (A) Expression of syncytin‐1 receptors ASCT1 and ASCT2 on MT‐2 cells was measured by flow cytometry. (B) A schematic diagram of the coculture protocol for HTLV‐1 transfer to trophoblasts. BeWo cells or PCTs were cocultured with MT‐2 cells (10:1 ratio of MT‐2 and target cells) for 48 h, followed by washing and further incubation. (C) Representative phase‐contrast images (scale bar = 200 μm) show reduced confluency in BeWo cells cocultured with MT‐2 cells. (D) Protein levels of HTLV‐1 p19, syncytin‐1, and β‐actin in BeWo cells and PCTs under basal conditions or after forskolin induction (20–50 μM for BeWo; 50–100 μM for PCTs), with or without HRB1‐mediated syncytin‐1 cell fusion blockade. The relative intensity of p19 expression is shown below with data normalized to the BeWo or PCT cocultured with MT‐2 cells without forskolin treatment. Syncytin‐1 expression was normalized to the corresponding group without forskolin treatment.

Syncytin‐1, a protein naturally expressed in placental trophoblasts, plays a critical role in mediating cytotrophoblast fusion to form syncytiotrophoblasts [30]. We investigated whether endogenously expressed syncytin‐1 facilitates cell‐to‐cell transfer of HTLV‐1 from T cells to placental trophoblasts. Primary cytotrophoblasts (PCTs), located in the inner layer of the villous trophoblast, are the stem cells of the trophoblast lineage. PCTs give rise to the more differentiated syncytiotrophoblasts and extravillous trophoblasts [31]. The human placental choriocarcinoma cell line BeWo, widely used as an in vitro placental model, was also employed [32]. For viral delivery, we used MT‐2 cells, an HTLV‐1‐infected T‐cell line with regulatory T‐cell characteristics [33], which expressed the syncytin receptors ASCT1 and ASCT2, as confirmed by flow cytometry (Figure 6A) [34].

PCTs or BeWo cells were cocultured with MT‐2 cells at a 10:1 ratio (MT‐2: target cells) for 2 days. After extensive washing with PBS to remove MT‐2 cells, the PCTs and BeWo cells were cultured for an additional 3 days before being harvested for protein analysis. (Figure 6B). Microscopic analysis demonstrated reduced confluency in BeWo cells cocultured with MT‐2 cells compared to controls by Day 5 post‐coculture (Figure 6C). Western blot analysis detected syncytin‐1 expression in both BeWo cells and PCTs, which was unaffected by coculture with MT‐2 cells. Strikingly, HTLV‐1 p19 was highly expressed only in target cells cocultured with MT‐2 cells, confirming viral transmission (Figure 6D).

To assess syncytin‐1's role in HTLV‐1 transmission, we employed HRB1, a syncytin‐1‐specific fusion inhibitor [35]. HRB1 pretreatment blocked cell fusion in the coculture system, as observed microscopically by Day 5 (Figure 6C). However, under basal syncytin‐1 conditions, HRB1 minimally impacted p19 expression in BeWo cells and PCTs. As forskolin upregulates syncytin‐1 in trophoblasts [36], we pretreated BeWo cells and PCTs with forskolin (20 or 50 μM for BeWo; 50 or 100 μM for PCTs) for 24 h. This pretreatment enhanced syncytin‐1 expression in both cell types. Notably, subsequent addition of HRB1 reduced HTLV‐1 p19 protein levels by 30%–70% in forskolin‐pretreated BeWo cells (20 and 50 μM, respectively) and by 30% in forskolin‐pretreated PCTs cocultured with MT‐2 cells (Figure 6D). These findings demonstrate that syncytin‐1‐driven cell fusion is critical for HTLV‐1 cell‐to‐cell transmission in placental trophoblasts.

## 4 Discussion

Our study elucidates the persistent HTLV‐1 infection within placental villi, revealing a low‐level viral presence in trophoblasts that suggests partial evasion of placental barriers. Despite this, intrauterine transmission remains rare in the absence of breastfeeding, as evidenced by the lack of seroconversion in most non‐breastfed infants [3, 37, 38]. In addition, while some other mechanisms may exist to impede its transmission, HTLV‐1 is predominantly in a latent infection state in vivo. Thus, an active HTLV‐1 infection of placental cells is likely limited.

A key virological trait of HTLV‐1 is its reliance on cell‐to‐cell contact for its transmission [39]. After infection, its replication entails clonal expansion in lymphocytes in the peripheral blood [40]. HTLV‐1 replication may be self‐restrained, characterized by high‐fidelity replication, resulting in high genetic stability with elevated immune escape [41, 42]. Our data demonstrated that HTLV‐1 infection in the placenta is largely latent. Thus, the infection of trophoblastic cells by HTLV‐1 is not as productive as in the peripheral blood.

Placental susceptibility to viral infections often arises from immune dysregulation [43], and HTLV‐1 persistence may disrupt the delicate inflammatory balance critical for a healthy pregnancy. Notably, 44.4% (4/9) of HTLV‐1‐seropositive mothers enrolled in this study reported prior SAB, aligning with a 2–3‐fold elevated SAB risk observed in Peruvian cohorts [44, 45]. While our small sample size precluded deeper mechanistic insights, this association underscores the need for expanded studies on HTLV‐1‐associated placental pathology.

Elevated IFNβ levels in HTLV‐1‐positive placentas are particularly concerning, as excessive IFNβ can trigger placental apoptosis [29], potentially compromising fetal viability. IFN‐induced transmembrane proteins, such as IFITM inhibit virus infection by preventing virus membrane fusion with cells and inhibiting the fusion of infected cells [43, 46, 47]. Paradoxically, IFITM1—a viral entry inhibitor—showed no differential expression, suggesting alternative pathways drive placental inflammation. While they are tightly regulated during healthy pregnancies [48, 49], dysregulation of cytokines such as IL‐4, IL‐6, and TNF may contribute to placental inflammation. HTLV‐1 proteins Tax and HBZ further perturb immune homeostasis: Tax induces pro‐inflammatory IL‐2 and IL‐15 [22], while HBZ amplifies TGFβ signaling to modulate regulatory T cells [50, 51]. The balance of the placental environment is essential for the proper health and growth of the fetus. Apoptosis in the placenta of HTLV‐1‐seropositive pregnant women has been demonstrated as a possible defense mechanism against transmission from mother to fetus [52]. The difference in mean mother's age at delivery, number of previous pregnancies, among mother's groups can impact the expression of inflammatory markers.

The presence of HTLV‐1 DNA was extended beyond the trophoblast layers and was detectable in deeper placental cell layers. Since HTLV‐1 can directly infect multiple host cell types, including immune cells, trophoblasts, endothelial cells, and fibroblasts [53], it is conceivable that alternative pathways may facilitate the HTLV‐1 entry to the placental trophoblasts [54]. This might not involve specific steps of the viral replication cycle but serves as an entry mechanism for HTLV‐1 into trophoblasts. We demonstrated that inhibition of syncytin‐1‐mediated cell‐to‐cell fusion prevented HTLV‐1 infection in trophoblasts in vitro, but only in BeWo cells and PCTs pretreated with forskolin. This pretreatment increased syncytin‐1 expression in both cell types. Because forskolin may also stimulate HTLV‐1 LTR activity during viral transmission [55], we only pretreated trophoblasts, that is, BeWo cells and PCTs with forskolin for 24 h and washed out prior coculture with MT‐2. Therefore, MT‐2 cells were not exposed to forskolin. We found that HTLV‐1 p19 was enhanced by forskolin but reduced by the syncytin‐1–specific fusion inhibitor HRB1, indicating that syncytin‐1 contributes to HTLV‐1 transmission from T cells to trophoblasts, analogous to its role in HIV infection of the placenta [54]. Nevertheless, unaddressed confounders—such as syphilis coinfection and gestational diabetes in asymptomatic carriers—warrant future investigation. And the small number of placentas in this study could be a limiting factor for our statistical analyses. Also, around one‐third of the infected mothers had comorbidities and coinfection status in this cohort, which could skew the results. Thus, a higher number of placentas is needed to make a more convincing interpretation of the results in the future.

Together, the physiological placenta is competent to block intrauterine HTLV‐1 transmission. However, rather than directly transmitting the virus to the fetus, the latent HTLV‐1 infection in the placenta leads to a persistent but low level of inflammation, potentially increasing the risk of SAB. Further studies are warranted to comprehend the mechanisms of SAB, which may help develop interventions for the treatment of MLHTLV‐1.

## Author Contributions

Jorge Casseb, Yuyang Tang, and Guochun Jiang conceived the initial concept. Victor Folgosi, George Souza, Sandy Teixeira, Carlos Apoliano, Fernanda Grassi, Jorge Casseb, Jerusa Smid, Michel E. Haziot, Rosa Maria N. Marcusso, Augusto C. P. de Oliveira, and Tatiane Assone helped to coordinate the clinic study, cohort recruitment, and tissue collection. Gabriela Prates did ddPCR, DNA/RNAscope, IHC, and Western blot. Xiaoyi Li performed Western blot and Flow cytometry analysis. Hongjie Chen reanalyzed the statistics. Yuyang Tang helped syncytin‐1 studies. Gabriela Prates and Jorge Casseb organized the initial data. Gabriela Prates and Guochun Jiang prepared the initial manuscript. Xiaoyi Li helped with the manuscript revision. Guochun Jiang finalized the data analysis, initial submission, and manuscript revision.

## Conflicts of Interest

The authors declare no conflicts of interest.

## Supporting information


**Supplementary Table 1:** Characteristics of 18 mother participants in this study.

## Data Availability

The data that support the findings of this study are available from the corresponding author upon reasonable request. However, the patient‐related clinic data may not be publicly available due to privacy or ethical restrictions.
