# Supplementary material for: Persistent Human T‐Lymphotropic Virus Type 1 (HTLV‐1) Infection in the Placenta of Pregnant Women
Source: J Med Virol. 2025 Sep 5;97(9):e70585. doi: 10.1002/jmv.70585 (PMC12412083; doi:10.1002/jmv.70585)
Supplement: Supplementary file 1 — Supplementary Table 1: Characteristics of 18 mother participants in this study. [file JMV-97-e70585-s001.docx]

**Supplementary Table 1. Characteristics of 18 mother participants in this study.**

| **ID** | **Infected or not** | **PBMCs HTLV-1 DNA proviral load** | **Age**  **(years)** | **Delivery** | **Previous pregnancy** | **Coinfections/Comorbidities** | **HTLV-1-Clinical outcome** | **Previous Miscarriage** | **Syncytin copies /µg** | **IFIT-M copies /µg** | **IFN-ɑ copies /µg** | **IFN-𝛽 copies /µg** | **IFN-γ copies /µg** |
| --- | --- | --- | --- | --- | --- | --- | --- | --- | --- | --- | --- | --- | --- |
| **CTRL-1** | **HTLV-1 (-)** | - | 29 | Vaginal | 1 | 0 | - | 0 | 30331 | 2694 | 5181 | 4979 | 0 |
| **CTRL-2** | **HTLV-1 (-)** | - | 32 | Vaginal | 0 | 0 | - | 0 | 328444 | 3391 | 2817 | 14241 | 0 |
| **CTRL-3** | **HTLV-1 (-)** | - | 18 | Vaginal | 0 | 0 | - | 0 | 15157 | 9146 | 1028 | 0 | 0 |
| **CTRL-4** | **HTLV-1 (-)** | - | 26 | Vaginal | 0 | 0 | - | 0 | 406193 | 921 | 2093 | 0 | 0 |
| **CTRL-5** | **HTLV-1 (-)** | - | 19 | Vaginal | 0 | 0 | - | 0 | 272644 | 325 | 0 | 0 | 343 |
| **CTRL-6** | **HTLV-1 (-)** | - | 35 | Cesarean without label | 0 | 0 | - | 0 | 625238 | 945 | 1018 | 0 | 0 |
| **CTRL-7** | **HTLV-1 (-)** | - | 24 | Vaginal | 0 | 0 | - | 0 | 460076 | 320 | 0 | 0 | 0 |
| **CTRL-8** | **HTLV-1 (-)** | - | 19 | Vaginal | 0 | 0 | - | 0 | 262090 | 0 | 0 | 340 | 0 |
| **CTRL-9** | **HTLV-1 (-)** | - | 18 | Vaginal | 0 | 0 | - | 0 | 5155 | 0 | 0 | 0 | 0 |
| **No. 1** | **HTLV-1 (+)** | <detect. limit | 19 | Cesarean without labor | 1 | Syphilis | AC | 1 | 56350 | 3636 | 3636 | 3969 | 11059 |
| **No. 2** | **HTLV-1 (+)** | 27 | 22 | Cesarean **with** labor | 1 | No | HAM | 0 | 216000 | 2363 | 2363,4 | 7211 | 10059 |
| **No. 3** | **HTLV-1 (+)** | <detect. limit | 40 | Vaginal | 4 | Gestational Diabetes | AC | 0 | 11611 | 6363 | 6363 | 3757 | 7999 |
| **No. 4** | **HTLV-1 (+)** | *Not quantified | 25 | Cesarean without labor | 2 | Gestational Diabetes | AC | 0 | 261700 | 1647 | 1647,553 | 7817 | 0 |
| **No. 5** | **HTLV-1 (+)** | *Not quantified | 33 | Cesarean without labor | 1 | No | AC | 0 | 136163 | 2081 | 2081,908 | 10756 | 0 |
| **No. 6** | **HTLV-1 (+)** | 90 | 34 | Cesarean without labor | 2 | No | AC | 1 | 432354 | 0 | 0 | 5044 | 0 |
| **No. 7** | **HTLV-1 (+)** | *Not quantified | 42 | Vaginal | 3 | No | AC | 1 | 71831 | 0 | 0 | 3798 | 0 |
| **No. 8** | **HTLV-1 (+)** | 9810 | 39 | Cesarean without labor | 1 | No | AC | 1 | 48276 | 0 | 0 | 363 | 0 |
| **No. 9** | **HTLV-1 (+)** | 6 | 35 | Cesarean without labor | 4 | Gestational hypertension | AC | 1 | 5441 | 0 | 0 | 335 | 0 |

HTLV screening was performed during pregnancy. All HTLV+ donors were Western Blot and PCR tested positive. Under detection limit: The proviral load was under the detection limit <50 copies/10^6^ PBMCs. *Not quantified: Pregnant women did not agree to collect peripheral blood for this test during recruitment. AC, asymptomatic carrier; HAM, HTLV-1-associated myelopathy; UN, undetectable. The mother of No. 2 received methylprednisolone.
